# Rapidity and Precision of Steroid Hormone Measurement

**DOI:** 10.3390/jcm11040956

**Published:** 2022-02-12

**Authors:** Shigehiro Karashima, Issey Osaka

**Affiliations:** 1 Institute of Liberal Arts and Science, Kanazawa University, Kanazawa 921-1192, Japan; 2 Department of Pharmaceutical Engineering, Faculty of Engineering, Toyama Prefectural University, Imizu 939-0398, Japan

**Keywords:** steroid hormone, immunoassay, mass spectrometry, radioimmunoassay, gas chromatography tandem mass spectrometry, liquid chromatography tandem mass spectrometry, matrix-assisted laser desorption/ionization

## Abstract

Steroids are present in all animals and plants, from mammals to prokaryotes. In the medical field, steroids are commonly classified as glucocorticoids, mineralocorticoids, and gonadal steroid hormones. Monitoring of hormones is useful in clinical and research fields for the assessment of physiological changes associated with aging, disease risk, and the diagnostic and therapeutic effects of various diseases. Since the discovery and isolation of steroid hormones, measurement methods for steroid hormones in biological samples have advanced substantially. Although immunoassays (IAs) are widely used in daily practice, mass spectrometry (MS)-based methods have been reported to be more specific. Steroid hormone measurement based on MS is desirable in clinical practice; however, there are several drawbacks, including the purchase and maintenance costs of the MS instrument and the need for specialized training of technicians. In this review, we discuss IA- and MS-based methods currently in use and briefly present the history of steroid hormone measurement. In addition, we describe recent advances in IA- and MS-based methods and future applications and considerations.

## 1. Introduction

**Figure 1:**
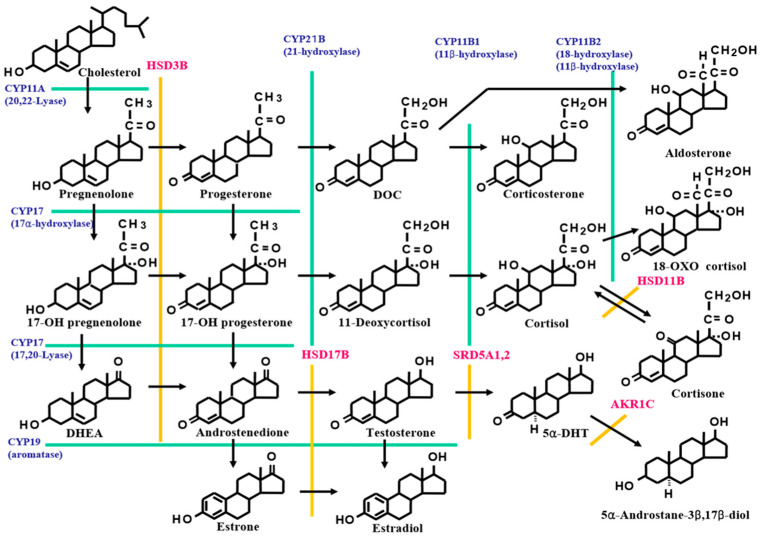
Structure and metabolic pathways of steroids. The steroid core structure is typically composed of 17 carbon atoms, bonded in 4 rings: three 6-member cyclohexane rings and one 5-member cyclopentane ring. Steroids vary in the functional group attached to this four-ring core and the oxidation state of the rings. The biosynthesis of steroid hormones begins with cholesterol as the starting material. First, high-density lipoprotein cholesterol in the blood is taken up into the cytoplasm and transported to the inner mitochondrial membrane via the action of steroidogenic acute regulatory protein (StAR), the cholesterol side chain cleavage enzyme, and other enzymes. Subsequently, 3β-hydroxysterone is synthesized, and various steroid hormones are biosynthesized via 3β-hydroxysteroid dehydrogenase, 17α-hydroxylase, 21-hydroxylase, and 11β-hydroxylase. All enzymes except 3β-hydroxysteroid dehydrogenase are cytochrome P-450 enzymes. 11β-Hydroxylase has 18-hydroxylase and 18-dehydrogenase activities, which are the final steps in the aldosterone synthesis system.

Steroids are compounds that are found in all animals and plants, from mammals to prokaryotes. Hormones with a steroid skeleton are called steroid hormones. The side chains attached to the steroid ring, which is the basic skeleton, have a variety of effects. Human steroid hormones are produced mainly in the ovaries, gonads, and adrenal cortex, using cholesterol as a precursor, and are classified as sex hormones, glucocorticoids, or mineralocorticoids based on their function. In clinical practice, estradiol, testosterone, cortisol, and aldosterone are often measured because they have high metabolic activity in vivo (Figure 1). Blood is a biological sample that is easily obtained clinically. Measurement of steroid hormone levels in serum and plasma is necessary to determine whether they are excessive or insufficient for homeostasis. In the current management of adrenal tumors, the measurement of steroid hormones is important for the functional diagnosis and postoperative evaluation of adrenal tumors. Immunoassay (IA)- and mass spectrometry (MS)-based assays are currently the main methods of steroid hormone measurement. The ideal assay system guarantees both rapidity and accuracy using a simple approach. Currently, IA is the most popular method in daily clinical practice because of its simplicity. However, liquid chromatography tandem MS (LC-MS/MS) and GC tandem MS (GC-MS/MS) yield more accurate steroid measurements than does IA.

In this review, we discuss the history of steroid hormone measurement and describe the immunoassay (IA) and MS methods currently used in medical practice. In addition, the accuracy and rapidity of the IA and MS methods, recent advances, and future applications and considerations are also discussed.

## 2. Early Methods for Steroid Hormone Analysis

The history of the measurement of steroid hormones began with their discovery and isolation. In the late 1920s, steroids were typically isolated from urine and gonadal tissues. However, the amount present was so small that it took a lot of effort to obtain a few grams of the substance. Estrone was the first steroid to be isolated [1,2]. Thereafter, substances with steroidal skeletons were found in many plants and animals [3,4,5,6,7,8,9], and clinical and basic experiments showed that their activities had significant effects on biological processes.

During the 1950s, several attempts were made to measure steroids using chemical reactions; for example, the Nelson-Samuels method for measuring cortisol in the blood is based on the so-called Porter-Silber reaction, a color reaction specific to the dihydroacetone structure of 17- and 21-dihydro-20-ketone of cortisol [10,11]. In addition, with the development of chromatography, methods for measuring trace amounts of dihydroacetone after separation of cortisol became successful and widely implemented [12]. These steroid hormone assays were not considered suitable for use in clinical practice because of the long times required to process large numbers of samples [13].

The basic principles of IAs were developed in 1960 by Berson and Yarrow [14,15]. However, steroid hormones have low molecular weights (less than 500 Da) and are not antigenic; thus, antibodies are not produced. The protein binding method using cortisol-binding globulin, which can assess hormone levels in the blood, was established by Murphy and colleagues for measuring steroid hormones [16]. In 1969, Abraham [17] used a radioimmunoassay (RIA) to measure blood levels of steroid hormones from generated antibodies for the first time. In the early 1970s, steroids such as cortisol and aldosterone could be measured by RIA [18,19,20,21,22,23]. In subsequent years, researchers developed RIAs for measuring other important steroid hormones in vivo. Thus, the establishment of RIAs made it possible to quantify steroids from small amounts of serum isolated from the peripheral blood, marking the beginning of a new era in endocrinology.

However, the beginnings of the history of MS predate that of IAs. In 1919, F.W. Aston developed the predecessor to the current MS instrument [24]. In the 1930s, deuterium was successfully isolated [25], and sterol metabolism was studied using labeled precursors [26]. Systematic electron impact fragmentation studies of steroids were performed, establishing the basis for structure determination. However, it was not until the 1960s that the combination of chromatography and MS made it possible to identify steroids from biological samples [27]. Eneroth et al. [28] reported the first paper on combined gas chromatography (GC)-MS of human sterols. GC-MS was widely used for clinical steroid analysis during the 1970s, and accurate quantitative methods utilizing labeled internal standards of many of the important compounds we measure today by tandem MS were established by Ingemar Björkhem and colleagues [29]. Table 1 summarizes the years in which steroid isolation and measurement methods were reported.

## 3. IAs

### 3.1. RIAs and Non-RIAs

There are two types of RIA methods: competitive and sandwich. In the competitive method, an antigen labeled with a radioisotope is mixed with an antibody targeting the antigen at a known dose, and an unlabeled sample of interest is then added to measure the amount of labeled antigen that is not bound to the antibody [35,36]. The sandwich method is used when the antigen binds to more than one antibody. First, the antibody is immobilized in the solid phase, and the antigen sample to be measured is then added and bound [37]. When another radiolabeled antibody is added, the label is detected in the solid phase depending on the amount of antigen. However, because of the complexity of the RIA procedure, radioactive isotope waste at the time of measurement, and maintenance of testing facilities, most modern clinical practices use nonisotopic IAs (non-RIAs).

Non-RIA methods include enzyme IAs [38], enzyme-linked immunosorbent assays [38,39], chemiluminescent immunoassays [40], and electrochemiluminescence immunoassays [41], which use enzymes and chemiluminescent substances as labeling materials instead of radioisotopes. Non-RIA methods have several advantages, including avoiding radioactive isotope exposure and contamination and greatly simplifying work procedures.

Despite these advantages, IAs also show problems with cross-reactivity of various steroids and a lack of unification of steroid reference materials for IA testing. Since human samples contain steroid compounds with similar structures, immunoassays are prone to cross-reactivity with substances other than the subject of interest. For example, Krasowski et al. [42] measured the cross-reactivity of structurally diverse compounds using Roche Diagnostics Elecsys assays for cortisol, dehydroepiandrosterone (DHEA) sulfate, estradiol, progesterone, and testosterone. The Elecsys cortisol and testosterone II assays showed a wider range of cross-reactivity compared with assays for DHEA sulfate, estradiol II, and progesterone II. 6-Methylprednisolone and prednisolone also showed high cross-reactivity in cortisol assays and were shown to be likely to have clinically significant effects in patients receiving these drugs [42]. In the first half of 2000, commercially available IA kits for total testosterone and free testosterone were used to detect low concentrations of the target substance in women, children, and testosterone-deficient men. However, reports have shown that accuracy cannot be guaranteed [43,44,45]. Similar reports have been published for assay kits detecting estradiol in children [46], men [47,48], postmenopausal women [49,50,51,52], and women on aromatase inhibitor therapy [53]. As a result, the Endocrine Society published position statements on the measurement of testosterone in 2007 [54] and estradiol in 2013 [55]. Both testosterone and estradiol are well suited for measurement under high concentration conditions that exhibit hormone overproduction; however, measurement of samples with low concentrations can be problematic. In addition to gonadal hormones, agreement between serum and salivary cortisol levels using commercial reagent kits was found to be unsatisfactory for cortisol [56,57]. Although some IAs have shown excellent agreement with the MS method [58,59], the majority of recent publications have demonstrated major nonlinearities between cortisol measurement by IA and MS, with a general lack of accuracy for different IAs [56,57,60]. In 2013, the *Journal of Clinical Endocrinology and Metabolism*, the flagship journal of the Endocrine Society, published an editorial on the requirements for steroid MS, where quality control of assays, including sensitivity and reproducibility assessments using MS, must be reported and presented in detail [61].

Furthermore, it is necessary to develop standard reference materials based on a “universal value” to compare and contrast clinical laboratory data. Quantitative test results must be verifiably traceable to a common reference material (certified reference material), if available. Information on the available reference materials can be obtained from the Centers for Disease Control and Prevention’s Clinical Standardization Programs [62]. Moreover, the National Institutes of Health has also developed a standard for steroid metabolomics research [63]. However, the reference materials for commercially based steroid assay kits vary among manufacturers, and this issue may also contribute to the observed variability in assay values. This limitation is also observed for MS-based assays. Indeed, even when high-purity steroid standards are available, improper storage and handling can alter the structure of steroid standards, resulting in conversion to other steroid compounds. Accordingly, certified reference materials for clinical testing need to be developed and used to price and evaluate product calibrators offered by each clinical reagent manufacturer.

### 3.2. Rapid IAs

The steroid assay kits available at most medical facilities are based on IAs, which require approximately 60–120 min to obtain results. Clinics that outsource steroid testing to outside laboratories may require 1–2 days to obtain results. Recently, two rapid non-RIAs have been reported, i.e., gold-nanoparticle-based immunochromatographic Quick Cortisol Assay (QCA) [64] and chemiluminescent enzyme IA for aldosterone (CLEIA) [65]. Yoneda et al. [64] developed a rapid cortisol measurement kit (Trust Medical, Kasai, Japan) that can be used during adrenal vein sampling (AVS), which is the gold standard for the local diagnosis of aldosterone in PA. With proof of aldosterone hypersecretion from one, but not both adrenal glands, a complete cure can be achieved by removal of the causative unilateral adrenal gland. However, the success rate of AVS has been reported to be low due to the thinness of the left and right adrenal veins and the wide anatomical variation among individuals.

Plasma cortisol levels are difficult to measure during AVS because they are measured over approximately 1 h per sample using a nonportable automated measuring device installed in the laboratory. To measure cortisol during AVS, it would be necessary to move specimens from the angiography room to the laboratory every time blood is drawn during AVS. In addition, it would be necessary to stop all blood tests collected in other clinics to prioritize the measurement of AVS specimens, which would be very disruptive to the practice. The low success rate of AVS (50–80%) is also associated with the termination of AVS before determining cannulation success or failure [66,67]. Notably, several groups have performed cortisol measurement during AVS and reported that intra-operative cortisol measurement during AVS increases the success rate of AVS [68,69,70,71,72]. However, there are many issues that must be addressed, such as the length of time required to perform AVS (e.g., several tens of minutes from sample collection to determine the results of cortisol concentrations) and the need for skilled technicians to perform the test.

**Figure 2:**
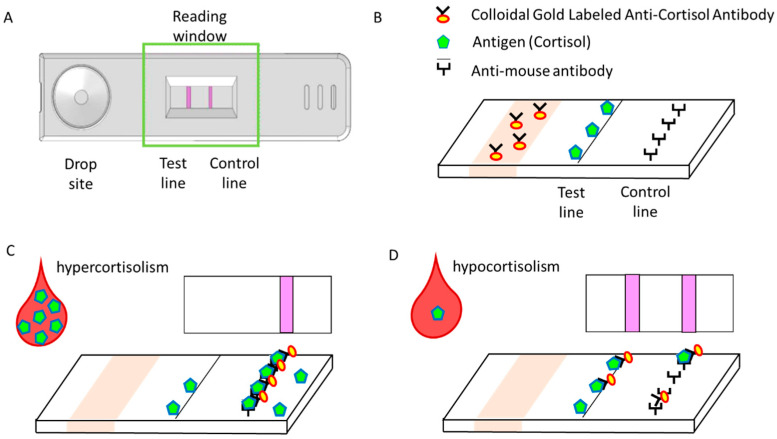
Quick cortisol assay based on immunochromatography and gold nanoparticles. (**A**) The strip consists of a drop site and a reading window. Plasma (100 μL) is placed in the well over the immunochromatographic paper strip. (**B**) Plasma applied to the drip site reacts with Colloidal Gold Labeled Anti-Cortisol Antibody (Ab·Gold). Cortisol (antigen: Ag) is present in the test line, and anti-mouse antibody is present in the control line. If plasma contains a sufficient amount of cortisol, the Ag-Ab·Gold complex is formed. Then, the complex migrates toward the reading window, and the complex is caught by the anti-mouse antibody. (**C**) Excess Ab·Gold and/or “Ag-Ab·Gol” complex migrates to the control line where anti-mouse IgG is impregnated. These molecules are bound by anti-mouse IgG at the control line. If the plasma cortisol level is high, only one magenta line appears, with no apparent test line. (**D**) If the cortisol content in the plasma is insufficient and/or is not present, two magenta lines appear at the test and control lines.

The QCA kit is capable of measuring cortisol levels in under 5 min (range: 5–30 μg/dL), which is required to determine successful cannulation during AVS without adrenocorticotropic hormone loading. Although the measurements are not automated, only a small reader and strip are needed. This is advantageous because AVS is typically performed in a small, densely populated angiography room, preventing the installation of a large instrument or a densitometer. This also eliminates the need to transport the specimen to the laboratory. Additionally, this kit is based on a competitive method using an anti-cortisol monoclonal antibody (Figure 2). The cortisol in a drop of serum binds to the anti-cortisol monoclonal antibody, which is labeled and immobilized on gold colloid. Upon further expansion on the membrane, the immobilized antigen (bovine serum albumin-labeled cortisol) reacts with the unreacted anti-cortisol antibody-sensitized gold colloid particles. By measuring the absorbance of the test line, cortisol levels can be quantified. In addition, the test line was designed such that it is not visible at cortisol levels greater than or equal to 30 μg/dL, enabling semiquantification. Before and after using the kit, the success rate of cannulation in AVS increases significantly from 63% to 93% [64]. Overall, the simplicity of the technique and the fact that the measurement can be performed in the angiography room without interrupting other tests in the clinical laboratory represent the substantial advantages of this approach in a clinical setting.

Morimoto et al. [65] applied an antibody-immobilized magnetic particle system, called MAGRAPID (Wako Pure Chemical Industries, Ltd., Osaka, Japan), to CLEIA technology owing to its high magnetic response and rapid dispersibility. The authors rapidly performed screening tests using this technology in patients with PA. After antibody binding to the surface of the magnetic particles to be used as the solid phase and specifically reacting with the target in the sample, the particles were washed several times to remove other impurities. The antigen reacts with the antibody in the solid phase, and the sample is then washed to remove the free enzyme-labeled antibody. The concentration of the target is determined using a chemiluminescent reagent. The CLEIA system can measure plasma aldosterone concentrations in the range of 50 to 1000 pg/mL in approximately 10 min, making it easy to use in general laboratories. The results obtained through this method also show a high correlation with aldosterone concentrations measured by LC-MS/MS. Moreover, Ozeki et al. [73] developed a new chemiluminescent enzyme immunoassay using a two-step sandwich method and reported a higher correlation with aldosterone concentrations measured by LC-MS/MS than by the conventional RIA method [73]. Notably, in-hospital measurement of aldosterone using the CLEIA method allows for same-day reporting of aldosterone results. Many clinics and hospital laboratories that outsource their aldosterone measurements to outside reference laboratories take several days to obtain results. Thus, the CLEIA method reduces the number of visits to the hospital for testing and greatly reduces the time required to decide on a treatment plan.

Overall, rapid reporting of test results to patients using these assays can shorten the time from diagnosis to treatment. Thus, rapid IAs are invaluable in situations where faster clinical decisions are required.

## 4. MS

### 4.1. GC-MS

Metabolomics with highly sensitive analytical method, MS, provides useful information in disease diagnosis [74]. It has been widely applied to discover new biomarkers for various diseases. A metabolomic approach for the detection of amino acids and acylcarnitines in blood using MS showed that gestational age, parenteral nutrition, and caffeine treatment affect the blood metabolome [75]. Metabolite levels in male mid- and late preterm infants were most significantly affected by caffeine. Furthermore, infection with the highly pathogenic and infectious SARS-CoV-2 coronavirus has been proven to affect metabolism, as shown using MS analysis [76]. COVID-19 has spread since 2019 and it is causing clinical sequelae and deaths. In the MS analysis of COVID-19 patients, the variations in the serum metabolome affect liver metabolism.

Initial steroid metabolomics was performed by GC. Then, GC-MS was recommended for the analysis. The GC-MS method is a mass spectrometric technique coupled with GC using capillary GC columns [77]. Many advances in GC-MS have been reported. Generally, electron ionization (EI) [78] is the ionization method employed for GC-MS. First, vaporized volatile molecules are subjected to GC and separated by a GC column. Second, separated molecules are ionized by EI, and the produced ions are detected by MS. In the EI process, the target molecule gas is exposed to electrons accelerated to 70 eV. The molecules lose electrons by impact with 70 eV electrons, and radical cations are then produced from the molecule. The excess of energy transferred from the accelerated electron to the molecules not only ionizes them but also causes their fragmentation. Additionally, it is also possible to analyze the detailed structures of the target molecule for evaluation of the obtained fragment ions in GC-EI-MS. In GC-MS analysis of steroids, steroids are derivatized for trimethyl-silylation because of the increasing volatility of steroids.

GC-MS analysis of steroids in urine was first performed in the 1970s. Owing to the low volatility of sulfate steroids or glucuronide conjugates, steroids in urine require derivatization and hydrolysis before GC-MS. Although GC-MS is associated with limited automation capability and increased extraction complexity, advances in the technique have enabled the measurement of up to 40 steroids [79]. For the analysis of steroids in plasma and serum, Santen et al. [53] developed a highly sensitive GC-MS/MS assay that allows for the detection of estradiol at concentrations as low as 0.63 pg/mL; estradiol levels in normal postmenopausal women and in women with breast cancer could thus be determined using the GC-MS/MS method. Hansen et al. [80] reported that their GC-MS/MS method enables the detection of 0.08–0.16 ng/mL estrogen (i.e., estrone, 17β-estradiol, and 17α-estradiol), 0.20–0.36 ng/mL androgen (i.e., dehydroepiandrosterone, androstenedione, testosterone, and dihydrotestosterone), and 0.36–0.43 ng/mL progestagen (i.e., pregnenolone and progesterone) in serum and plasma simultaneously. Using this method, a two-step solid-phase extraction (SPE) procedure for sample pretreatment is required to achieve the limit of detection (LOD). Moreover, a GC-MS/MS method was developed for the simultaneous detection of 17 steroid hormones in human plasma. The obtained LOD was below 1 ng/mL for the analysis of derivatized steroids with N-methyl-N-trimethylsilyl-trifluoroacetamide [81]. Verification of the stability of steroid is required for the reliable sampling procedure and measurement. There are not only steroids, but also steroid conjugates in vivo. In urine, it has been reported that the amount of each steroid depends on the deconjugation reaction of the steroid conjugates [82]. The deconjugation and degradation conditions were in the order of pH values of 9 > 7 >> 5 and temperatures at 37 °C > 25 °C > 4 °C >> −20 °C, respectively. Due to the above reason, samples containing the steroids should be stored at −20 °C and pH 5–7. The temperature stability tests of steroids have also been evaluated by LC-MS [83]. Stability experiments of dehydroepiandrosterone sulfate (DHEAS), epiandrosterone sulfate (EpiAS), androsterone sulfate (AS), testosterone glucuronide, and testosterone sulfate showed no degradation effects in the steroid profile of samples stored for long periods (>10 years) at −20 °C.

The sample stability determines the time limit required from sample collection to analysis. Therefore, the advancement of technologies and the discovery of compounds with a long detectable time are required. In the measurement of anabolic androgenic steroids (AASs) analyzed with high frequency in previous years, the conjugated steroids are often used as target analytes for the steroids conventionally. In a previous report, sulfated metabolites were found to have a long detectable time [84]. Simultaneous analysis of the sulphated AASs and the other mandatory targets was proposed using full scan high resolution GC- low energy electron ionization (LEEI)-MS, with the sample procedure consisting of two liquid-liquid extraction (LLE). In the LLE, ethyl acetate for non-hydrolyzed steroids and methyl *t*-butyl ether (MTBE) for hydrolyzed steroids were used as the extraction solvent. They were combined afterwards and analyzed.

For current steroid analysis, the GC-MS/MS method is not widely used but does show high sensitivity for steroids, likely because of the long time required for the measurement and derivatization process with N-methyl-N-trimethylsilyl-trifluoracetamide [85]. On the other hand, direct detection of nonvolatile steroids without derivatization and reduced measurement times are possible using LC-MS/MS. Therefore, LC-MS/MS has become the most commonly used analytical method for steroid measurement. However, GC-MS is still sometimes used. Therefore, new review articles summarizing new analytical methods for GC-MS and LC-MS is important for the analysis of mandatory steroids containing sex hormone [86,87,88]. This review discusses the current analytical methods to evaluating steroid changes in steroidomics and steroid pathways [86].

### 4.2. LC/MS/MS

Currently, human serum and urine metabolomics are generally performed using high throughput LC-MS/MS [89]. LC-MS/MS is an analytical technique that combines high-performance liquid chromatography (HPLC) with highly sensitive and selective tandem MS. Electrospray ionization (ESI) is typically applied as the ionization method for LC-MS/MS. Conventionally, a triple quadrupole mass spectrometer with high sensitivity and a wide dynamic range has been used for quantitative analysis in LC-MS/MS [90,91]. Recently, many quantitative analytical methods have been reported, using an orbitrap mass spectrometer [92,93] owing to the improved detection sensitivity and dynamic range of this instrument.

Biological samples, such as plasma, serum, and urine, contain many matrix components. When these samples are simultaneously analyzed with target molecular ions in MS without HPLC, the sensitivity decreases, owing to the ion suppression effect of the matrix [94,95]. To prevent this effect, the matrix and target molecules are first separated by HPLC, and the ions are then detected by ESI-MS/MS in LC-MS/MS. However, it is difficult to completely separate the target molecules and all matrix components using HPLC because of the large amount of matrix in biological samples. Therefore, biological matrices in the sample are often removed using pretreatment methods such as deproteinization [96], LLE [97], and SPE [98,99] before high-sensitivity quantitative analysis by LC-MS/MS. Indeed, in a previous report, the recoveries of 50 pg DHEA and 0.5 pg 17β-estradiol in plasma were 104% and 107% using SPE, respectively [99]. For the urine sample, a solution containing 39 steroids eluted from PRiME HLB cartridge was dried and reconstituted with 100 μL methanol. It was then analyzed by LC–MS/MS with high sensitivity [100].

In the triple quadrupole mass spectrometer, ions of the selected target molecules among the ions generated by ESI are extracted by the first quadrupole (Q1) [101]. The ions extracted by the Q1 are collided with Ar gas and fragmented by the second quadrupole (Q2) [102,103]. The fragmentation method is called collision-induced dissociation (CID). The ion with a high ion yield among the obtained fragment ions by CID is specified. The ion is extracted by the third quadrupole (Q3) and detected. This method of analysis is called selected reaction monitoring (SRM), whereas SRM for the analysis of multiple product ions is referred to as multiple reaction monitoring (MRM; Figure 2).

In MRM for LC-MS, five steroids (testosterone, progesterone, cortisol, cortisone, and dihydrotestosterone) are detected with LODs of 0.4, 0.4, 1.9, 0.3, and 1.4 ng/mL, respectively [90]. However, in MRM, the number of target molecules depends on the scanning rate of MS and the channel limit for the software, and all target compounds and their fragmentation patterns must be known before measurement. It is difficult to carry out MRM with quadrupole time-of-flight (Q-TOF) and Q-Orbitrap MS, because these approaches cannot select and extract ions in TOF and orbitrap mass spectrometers. Nevertheless, these approaches have been used for the quantitative analysis of steroids without ion extraction at the Q3 after ion extraction at the Q1. This approach allows for highly sensitive detection because Q-Orbitrap and Q-TOF have a high resolution and selectivity, achieving high accuracy [92,93]. Using Q-Orbitrap MS, the LODs of progesterone, testosterone, cortisol, and androstenedione in human plasma are approximately 39.1 pg/mL [93]. Recently, the approaches of quantitative analysis using orbitraps has been increasing. Because Q-TOF mass spectrometers have a higher scanning speed than triple quadrupole, Q-TOF have recently been used for nontarget analysis of steroid compounds without extraction of the target ion alone [104,105]. The nontarget analysis could allow to detect both expected and unexpected compounds since it could detect all ionized molecules. In the nontarget analysis of samples from 19 healthy male volunteers with different genotypes of the UGT2B17 enzyme responsible for glucuroconjugation of testosterone, the LODs of testosterone and epitestosterone were both approximately 0.5 ng/mL. Congenital adrenal hyperplasia (CAH) can be diagnosed before symptoms appear using newborn screening. However, traditional screening by EIA results in a large number of false positives. To overcome this problem, Schwarz et al. [106] reported a method for identifying infants with CAH by analyzing 17-hydroxyprogesterone, androstenedione, and cortisol using LC-MS/MS.

**Figure 3:**
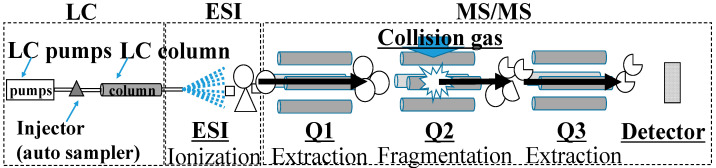
Liquid chromatography tandem mass spectrometry (LC-MS/MS) with multiple reaction monitoring. Components in the sample are separated by high-performance liquid chromatography (HPLC) and ionized by electrospray ionization (ESI). The target ions are extracted by the first quadrupole (Q1). Next, the extracted ions are collided with the collision gas, e.g., argon, nitrogen, or helium, in the second quadrupole (Q2). The ions with high ion yield among the obtained fragment ions are extracted and detected.

In LC-MS/MS, target molecules can be identified and quantified using three labels: (1) the *m*/*z* value of the target molecular ion associated with the first QMS, (2) the *m*/*z* value of the fragment ions associated with the third QMS, and (3) the retention time associated with HPLC (Figure 3).

Practical applications of LC-MS/MS have been reported. In our previous works, we established a method for the simultaneous analysis of 16 steroid hormones containing sulfide steroids using LC-MS/MS [107,108]; a *KCNJ5* mutant harboring a causative gene mutation of aldosterone-producing adrenal adenoma was prepared. Steroid metabolomic analysis of cultured cells with the *KCNJ5* mutation was performed and compared with that of the human adrenal carcinoma cell line (HAC15) without the *KCNJ5* mutation [107]. This experiment demonstrated that the presence or absence of the *KCNJ5* mutation dynamically regulates not only aldosterone production but also the entire steroid metabolic pathway. Comprehensive steroid analysis using LC-MS/MS is promising for the development of steroidology.

There are three problems that hinder the standardization of steroid measurements by LC-MS/MS. First, mass spectrometers are complex and delicate; therefore, expert inspection technicians are essential for the maintenance and establishment of measurement systems. Education in the inspection department of a medical university is insufficient because MS engineers are required to understand gas-phase organic chemistry for ion fragmentation in MS/MS, optics, and electromagnetism for ion behaviors in the mass spectrometer, and oxidation-reduction reactions for ionization to ensure analytical quality control. This is because the detailed principles of ionization remain unclear. There are many books on MS of organic compounds; however, no single author had compiled a comprehensive treatise including basic principles of MS until recently. Gross compiled the basic principles of mass spectrometric methods and applications of qualitative and quantitative analysis; nevertheless, basic knowledge of chemistry and electromagnetism is required to understand this information. The third edition of this book was published in 2017 [109].

The ESI technique used in LC-MS/MS was developed by Dr. Fenn, who shared the Nobel Prize for Chemistry in 2002 [110]. In our previous works, we investigated the relationships between the physical properties of peptides and ion yields by ESI [111]. The results showed that compounds with high ion yields in ESI-MS had a slight polarity and a high degree of hydrophobicity. However, because the principle for the reactions in charged droplets produced by ESI and in ion extraction to gas phase from such droplets has not yet been clarified, interpretation of the results often relies on experience with MS measurements. Previously, atmospheric pressure chemical ionization (APCI) was used as an ionization method for LC-MS of steroids [112]. However, the ionization efficiency of APCI is not always high for steroids, and thus, atmospheric pressure photoionization (APPI) was performed in attempts to increase this efficiency [113]. In LC-APPI-MS, it is necessary to carefully select the dopant compounds for ionizing steroids. Taking into consideration photon energy and the ionization energy of solvent and dopant molecules is also important. Currently, ESI is the most commonly used technique for steroid analysis because it is relatively simpler to understand the ion species obtained by ESI than those obtained by APCI and APPI. Thus, to fully exploit the performance of MS, it is necessary to have knowledge of chemistry and electromagnetism related to the physical properties of the compound, composition of the HPLC mobile phase, ionization and desorption efficiency, and relationship between the optics of MS and gas-phase organic chemistry to understand the fragmentation of molecular ions in MS.

Second, it is difficult to prepare high-purity steroid standard samples. LC-MS/MS is a highly sensitive analytical method that can detect steroids at concentrations as low as 10 pg/mL following injection of low volumes (10 μL) of plasma or serum. Generally, steroid standard reagents that can be purchased have a purity of 95–99%, and steroid synthesis and extraction can result in an error of approximately 0.3%. Additionally, impurities, such as phthalate compounds eluted from plastic sample tubes and lids during sample preparation, may also be identified [114,115]. Thus, these contaminants can affect the LC-MS/MS data and alter the structures of steroids during storage. Therefore, knowledge of organic chemistry is required.

The third problem is the associated costs. Steroids that are commonly evaluated in the clinic include cortisol, aldosterone, and a few gonadotropins. However, outsourcing of LC-MS/MS analysis is expensive. If all types of classical steroid maps could be measured, the cost may be lower than that of traditional IA methods. However, it takes training to accurately analyze and interpret all steroid data, including metabolic processes and metabolites, for a given condition.

Because nontarget LC-MS provides enormous amounts of information, it may also be difficult to analyze all data in detail. Recently, analyzing MS data in detail has become possible using statistical processing technologies, such as principal component analysis (PCA) [116,117,118,119], and accumulation of LC-MS data to identify the causative factors and novel biomarkers of diseases. Wawrzyniak et al. reported significant differences in metabolites containing lipids, amino acids and fatty acids between resistant and effectively controlled hypertensive patients [116]. Serum steroid hormone profiling can help establish a diagnostic approach for prostate cancer. Indeed, Albini et al. [117] evaluated the steroid profiles of 71 serum samples (31 controls, 20 patients with prostate cancer, and 20 patients with benign prostate hyperplasia) using PCA with LC-MS and found a clear and significant separation of prostate cancer with false negatives and benign prostate hypertrophy.

To accurately elucidate steroid profiles in serum, fluctuating levels of four steroids (estradiol, progesterone, cortisol, and testosterone) during the estrous cycle of canines were compared across three experimental groups using LC-MS/MS with multivariate statistical analysis. The results showed that the concentrations of these steroids exhibited characteristic patterns for each group at each specific estrous phase [118]. Thus, the PCA method can integrate complex information of multiple components in a sample and group them according to sample characteristics. Accordingly, LC-MS/MS combined with PCA may have many applications for medical science.

Recently, analysis of glucuronide and sulfate metabolites of seven anabolic-androgenic steroids AAS in urine using LC-MS with field asymmetric waveform ion mobility spectrometry (LC-FAIMS-MS) was reported [120]. Separation by the FAIMS-MS was investigated for selected cationic adducts. The method gave good reproducibility (RSD < 10%) and linearity (R^2^ > 0.99) in the range 3–20 ng/mL. Although the detection sensitivity is not enough, it is possible to suppress interference from the matrix. It is expected that the detection sensitivity will be improved in the future.

### 4.3. Matrix-Assisted Laser Desorption/Ionization (MALDI)-MS Imaging

**Figure 4:**
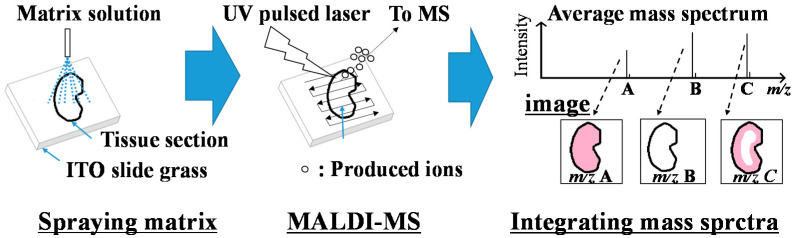
Overview of matrix-assisted desorption/ionization mass spectrometry (MALDI-MS) imaging. First, a MALDI matrix solution is sprayed onto mounted tissue sections on indium tin oxide (ITO) glass slides. Then, the sample is irradiated with a UV pulse laser in the MALDI mass spectrometer, and the ions produced from tissue sections are detected. Finally, images of the detected ions on tissues are integrated with the obtained MALDI mass spectra.

Koichi Tanaka at the Shimadzu Corporation, who shared the Nobel Prize for Chemistry in 2002, developed a laser desorption/ionization method that is the basis for MALDI [121]. In MALDI, a mixed crystal of a target sample with an ultraviolet (UV)-absorbing organic compound called a matrix, such as α-cyano-4-hydroxycinnamic acid and 2,5-dihydroxy-benzoic acid, is prepared, and then samples in the crystal are ionized via irradiation with a UV pulsed laser [122]. This method has been widely utilized for the analysis of proteins, low-molecular-weight biomolecules, and polymers. During sample preparation, it is difficult to precisely control the production of mixed crystals of the matrix and sample. Therefore, unlike LC-MS, MALDI-MS does not enable high-precision quantitative analysis. However, MALDI-MS is a powerful analytical tool that can be employed for highly sensitive qualitative analysis and semiquantitative analysis. Notably, MALDI-MS can yield localization images of components in tissue sections to integrate all mass spectra obtained by MALDI-MS recorded at regular intervals (Figure 4) [123]. This technique for constructing an image of a section after measurement by two-dimensional MS is called MALDI-MS imaging.

The sensitivity of MALDI-MS depends on the physical properties of the matrix and target substance [124]. Since a MALDI matrix that is compatible with all compounds in MALDI has not yet been developed, some compounds cannot be detected or only give a weak signal. Indeed, it can be difficult to detect steroids directly using MALDI, and derivatization with Girard’s reagent may be required [125]. To analyze the localization of steroids in tissue sections by MALDI-MS imaging, sections are sprayed with Girard’s reagent solution to derivate steroids and then sprayed with a MALDI matrix solution for MALDI-MS analysis.

PA is a disease caused by excess aldosterone. Aldosterone, a factor in PA, has the same mass as cortisone. Thus, MS/MS may be required, and MALDI-MS may not be sufficient without coupling with on-line HPLC. However, MS/MS of derivatized aldosterone and cortisone has been reported to yield different signals [126]. In a previous study, MALDI-MS/MS imaging was performed to distinguish and analyze the isomers aldosterone and cortisone. Although the serum marker of aldosterone-producing adenoma remains unknown because of poor techniques for adrenocorticosteroid visualization in tissues, aldosterone and 18-oxocortisol were found to co-accumulate within CYP11B2-expressing lesions [127]. Moreover, Cobice et al. [128] performed localization analysis of testosterone and 5α-dihydrotestosterone in mouse testes using MALDI-MS imaging; the LOD was less than 0.1 pg for testosterone derivatized using Girard’s T reagent.

Importantly, the reproducibility of MALDI imaging, as described above, varies depending on skill, technique, and experience. For example, it is important to achieve uniform coating of fine organic matrix crystals during pretreatment to obtain reproducible results. However, because it is difficult to spray the matrix and evaluate the uniformity and particle shape of its crystals, experience and skill are required. In addition to the general spraying method, a sublimation method [129] and a two-step matrix application method [130] coupled with sublimation and ultrasonic nebulization, have been reported for obtaining uniform crystals of the matrix on the structure surface. Laser desorption/ionization methods that do not use a MALDI matrix of organic compounds have also been reported [131,132]. Sunner et al. [131] developed novel laser desorption/ionization using graphite particles, called surface-assisted laser desorption/ionization (SALDI) because of the utilization of the material surface. Subsequently, SALDI using Au nanoparticles was reported by McLean et al. [132], and laser desorption/ionization with cobalt powder was also reported as a SALDI approach by Tanaka et al. [121].

**Figure 5:**
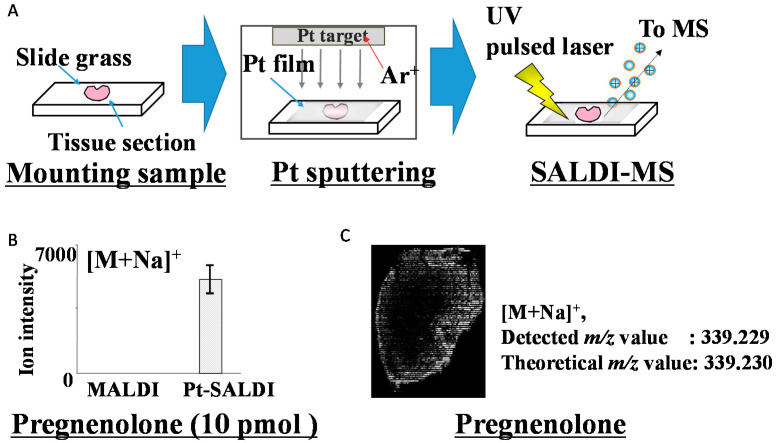
Surface-assisted laser desorption/ionization mass spectrometry (SALDI-MS) with a metal film. (**A**): Overview of SALDI-MS. Tissue samples are first cut using a microtome to obtain tissue sections and then mounted on glass slides. The samples are then coated with platinum (Pt) film using the sputtering method. Finally, the Pt film is irradiated with a UV pulsed laser, and the ions produced from the sample are detected. (**B**): Ion intensity of pregnenolone by MALDI and SALDI-MS. The average ion intensities are shown for MALDI and SALDI with Pt film (Pt-SALDI). Error bars represent the means ± standard deviations of five experiments. (**C**): Localization of [M+H]^+^ ions as a mass image of pregnenolone at *m/z* 339.229 obtained by SALDI-MS imaging of ferret adrenal glands.

The ionization ability of the method depends on the material species and shape, and may differ from that of MALDI. We previously developed a novel SALDI-MS imaging technique using a metal film [133,134,135] (Figure 5). In contrast to MALDI, SALDI does not require spraying of the matrix because of the direct preparation of uniform metal film on the sample surface using the sputtering method. A platinum (Pt) film also allows the direct detection of steroids without derivatization. Indeed, pregnenolone, which is associated with estrogen production, was directly detected without a derivatization procedure, and the localization of pregnenolone ions in ferret adrenal glands was obtained as a mass image (Figure 5C), demonstrating localization in the adrenal cortex. A thin Pt film is prepared by automatic and precise sputtering, obtaining a uniform film that can be easily prepared with a thickness on the nanometer scale and negating the need for specially trained technicians. Overall, establishment of an MS method that provides highly accurate data with simple pretreatment procedures, such as SALDI-MSI, can contribute to the elucidation of the roles of steroids in vivo.

## 5. Conclusions

In this review, we outlined the history, challenges, and IA- and MS-based principles of steroid measurement. In steroid assays, MS and IA have a complementary relationship. The selection and development of steroid hormone assays must focus not only on the quality of the assay but also on the intended use, such as whether specificity or rapidity is the priority. Economic considerations must also be taken into account; in small laboratories, MS is not cost effective, and clinical diagnosis will likely rely on IA. Although some experts are pushing for MS to become the mainstream method for measuring steroids, it would be unrealistic to expect IA, which is already in widespread use, to become obsolete. A well-validated and superior IA is also not inferior to MS. Thus, current assay selection should be judged by the performance of the assay in response to clinical need, not by assay technology. Accordingly, researchers and clinicians should be aware of the advantages and disadvantages of IA and MS to make the appropriate choice. It is possible that a new technique will be developed in the future that provides simple, rapid measurements similar to that of IA but with the superior specificity of MS. Until then however, researchers will continue to improve upon IA and MS to overcome the problems associated with each technique.

## Figures and Tables

**Table 1:** Summary of reporting years for steroid isolation, immunoassay, and mass spectrometry measurements.

Steroids	Isolation	IA (RIA)	MS
Estrogens	Estrone in 1929 [1,2]	1971 [18]	1973 [30]
	Estradiol in 1935 [3]		
Androgens	Androgen in 1931 [4]	1970 [19]	1974 [31]
	Testosterone in 1935 [5]		
Progesterone	1934 [6]	1971 [20]	1974 [31,32]
Cortisol	1930 [7]	1972 [21]	1974 [33]
Aldosterone	1954 [8,9]	1970 [22]	1973 [34]

## Data Availability

Not applicable.
